# Book Reviews

**Published:** 1823-08

**Authors:** 


### CRITICAL ANALYSIS
### OF
### ENGLISH AND FOREIGN LITERATURE
### RELATIVE TO THE VARIOUS BRANCHES OF
### Medical Science.

---

Quæ laudanda forent, et quæ culpanda, vicissim

Illa, prius, cretâ; mox hæc, carbone, *notamus*.—PERSIUS.

---

### DIVISION I.

### ENGLISH.

ART. I.—*A Treatise on Mental Derangement. Containing the Substance of the Gulstonian Lectures, for May* MDCCCXXII. By FRANCIS WILLIS, M.D. Fellow of the Royal College of Physicians.—8vo. pp. 234. Longman and Co. London, 1823.

THE name of WILLIS has become so much associated with the subject of the treatise before us, that we naturally sit down to its perusal with favourable anticipations. A general idea of the plan may be conveyed in few words: it consists in some remarks on the nature and origin of derangement; description and method of cure of the *high* and *low* states; and a concluding chapter on lunacy.

The object of the first chapter is to consider the state of man when his mental faculties are unimpaired, and to examine the connexion between the mind and body, with a view of showing that derangement cannot be remedied by means affecting the former alone, but requires the co-operation of the usual therapeutic agents. To illustrate the pernicious tendency of a contrary doctrine, the author informs us that he has heard a lecturer give it as his opinion that *furor uterinus* was a disease exclusively of the mind, and therefore not to be cured by medicines; quoting, in confirmation, the words of Macbeth,

" Can'st thou not minister to a mind diseased?"

Our author here takes occasion to descant at some length on furor uterinus and the tragedy of *Macbeth;* although we must confess we think the refutation of an opinion in itself so utterly untenable, and supported by a quotation so inapt, scarcely merited the pains bestowed upon it.

Some argument is next entered into, and supported by quotations from Gaubius and Cullen, to prove that the nervous system is the medium of communication between the body and mind. We pass over the discussion of a position which we imagine none of our readers will be disposed to call in question.

"These facts, I think, will make it evident that, for the reception of a true representation of any external object, or, in other words, for the transmission of a correct and faithful impression on the mind, the nerves should be in a perfectly healthy and natural state. We may infer this from diseases of the body, such as palsy and epilepsy, which, by disturbing the nervous system, never fail to weaken the mind. In every case of palsy the mind is somewhat affected, and, generally speaking, its affection keeps pace with that of the body. As the one is weakened and put out of order, so is the other. A man of the strongest mind, best understanding, and most quiet disposition, may, in a moment, in addition to the loss of power and motion, be bereft of these qualities: he is then incapable of connecting his ideas; his observations are childish; he alternately laughs and cries; his temper becomes irritable; his countenance idiotic: but, as his health improves, his mind returns to its natural state."

On the other hand, it is equally obvious that mental affections influence, to a great extent, the functions of the body. "The body and mind (says Gaubius,) are so conjoined in man, that, when both are in tranquillity, and in their most natural state, they seem to carry on their mutual commerce in some parts only: but, as often as there happens any considerable agitation, any great change in either of them, that change will sooner or later affect the other part, and by degrees spread itself over the whole man." Our author, in illustration, describes the state of an individual of regular habits, and unaccustomed to business, who becomes suddenly involved in anxiety and occupations, which deprive him of his usual relaxation and repose. During the continuance of the circumstances we have supposed, he becomes irritable, losing the usual command over his feelings and temper; "doing and saying many things from which at another time he would have refrained." Let him again return, however, to his former pursuits, and enjoy his usual rest, the symptoms will gradually disappear. But if, instead of this, the state of excitement be kept up for several weeks in succession, the symptoms become confirmed and increased, one step only is now required to constitute derangement,—viz. "his mind being impressed with false ideas, which will soon control all his thoughts and actions." As we are more capable of intellectual exertion when the body and mind are both alike free from fatigue, and as the exhaustion of either prevents us from being able to fix the mind in thought, so our author conjectures that such continued state of anxiety may operate upon the common medium of intercourse, and deprive the nerves of that peculiar state which may be called "tone." However this may be, it is certain that no one can have his mind long and closely occupied on any subject, without the risk of exciting confusion in his ideas, which, being continued, may end in derangement; and that his restoration can only be effected by a discontinuance of these exciting causes.

The same effects sometimes result from mental affections of much shorter duration, provided they be of considerable violence. Thus fright may lead to the firm belief of sounds, and even objects, which exist only in the imagination. "A young girl, (says Dr. Willis,) living in the same house with a patient in a raving delirium, became so terrified, that she one night called up her landlady, under the impression that the patient had escaped out of his room, and appeared at her bedside: she afterwards fainted away."

This illustration agrees with the opinions of Ferriar, who regards the stories of ghosts, and other "noctural illusions," as symptoms of bodily disease, as much as head-ach or shivering.

The second chapter is on the description of the *high state;* before entering upon which, the author premises some general observations. We are informed that there are two states of derangement which are to be considered; "both of them may in their progress pass into delirium, and again subside into derangement, and both, by neglect and improper treatment, may end in insanity; so that derangement, delirium, and insanity, are to be regarded as different degrees of mental disorder." The distinction between the three has been drawn by the distinguished uncle of our author,* in his examination before a committee of the House of Commons, in the following words:

"In *delirium*, the mind is actively employed upon past impressions, upon objects and former scenes, which rapidly pass in succession before the mind, resembling, in that case, a person talking in his sleep; there is also a considerable disturbance in the general constitution, great restlessness, great want of sleep, and a total unconsciousness of surrounding objects. In *insanity*, there may be little or no disturbance apparently in the general constitution; the mind is occupied upon some fixed assumed idea, to the truth of which it will pertinaciously adhere, in opposition to the plainest evidence of its falsity; and the individual is always acting upon that false impression. In insanity, also, the mind is awake to objects which are present. Taking insanity, therefore, and delirium, as two points, I would place *derangement of mind* somewhere between them."

The various forms of mental disease pass so insensibly into each other, as to become blended like the shades of light and darkness: we therefore cannot but regard all attempts to fix the boundaries of each by definition as altogether futile. The frequent allusion to SHAKSPEARE in the work before us, brings to mind the definition put into the mouth of Polonius by this the noblest of all our bards:

---

* Dr. R. D. Willis.

"Mad call I it; for, to define true madness,
What is't, but to be nothing else but mad?"

The description of the "high state" is taken from Dr. Munro's answer to Dr. Battie, and is so true to nature that most of our readers will at once recognise its fidelity: "High spirits, as they are generally termed, are the first symptoms of this kind of disorder; these excite a man to take a larger quantity of wine than usual: for those who have fallen under my observation in this particular have naturally been very sober, and the person thus afflicted, from being abstemious, reserved, and modest, shall become quite the contrary, drink freely, talk wildly, obscenely, swear, sit up till midnight, sleep little, rise suddenly from bed, go out a hunting, return again immediately, set all his servants to work, and employ five times the number that is necessary: in short, every thing he does or says betrays the most violent agitation of mind, which it is not in his own power to correct; and yet, in the midst of all this hurry, he will not misplace one word, or give the least reason for any one to suppose he imagines things to exist that really do not, or that they appear to him different from what they do to other people. They who see him but seldom admire his vivacity, are pleased with his sallies of wit and the sagacity of his remarks: nay, his own family are with difficulty persuaded to take proper care of him, until it becomes absolutely necessary from the apparent ruin of his health and fortune." Dr. Willis remarks, that, if the person here described had been a man of literary turn he would have been occupied in arranging and re-arranging his library; if a tradesman, he would probably have ordered goods of every one he met, without wanting them; and we might add, that, if a worthy citizen possessed of a seat at Islington, he would take the trouble to pull down his house, to build it on some better plan,—a species of derangement by no means uncommon. These symptoms of hurry and confusion are but the prelude to others of a more formidable nature: his irritability goes on increasing, and he at length becomes firmly persuaded in the reality of some visionary ideas. To constitute derangement, this state of mental alienation must be accompanied with bodily indisposition; otherwise, the case borders upon insanity, and the chance of recovery is considerably lessened.

"If, on the other hand, the symptoms of bodily indisposition increase, delirium ensues; and then the patient 'begins to rave, and talk wildly and incoherently; swears, as if in the most violent rage; and then, immediately after, bursts out into fits of laughter; talks obscenely; directs offensive and contemptuous language against his relations and those around him; spits at them; destroys every thing that comes in his way; emits loud and discordant screams, and continues in this way till he is quite exhausted. The state of rest which follows is generally short and sleepless. The patient is obstinate, will not speak one word; clenches his teeth if any thing is offered him to swallow; or else, with a degree of cunning, he pretends to drink a little, but immediately squirts it out again on the person who offered it. At once, however, he again breaks out into all the wild and extravagant language and actions he committed before. If kept in strict coercion, he has often so much command over himself as to behave mildly and modestly; and, were it not for the general expression of his countenance, and the peculiar glistening appearance and rapid movement of his eyes, he might impose on many of the by-standers, and make them imagine that the state of phrensy was over.'

"This is by no means an exaggerated representation. The symptoms of the complaint may vary in every individual. In some, the irritation is never so great as to end in delirium. When it does, the paroxysm continues for a longer or shorter period in different cases: in some instances, for only a few minutes; in others, the patients talk incessantly, for twenty, thirty, forty, or fifty hours, and then cease, apparently from exhaustion. After an hour's rest, they begin again more violently than ever, particularly if they should have fallen asleep; for this scarcely ever benefits the mind at first, but only recruits the body, enabling the patients to exert themselves with still greater vigour. During this state, we find that women are more prone than men to talk obscenely; the most modest of them will utter the most indecent expressions. This circumstance even Shakspeare has noticed in his character of Ophelia. Many exhibit talents which they do not possess when in health; and which, being foreign to their disposition, gradually forsake them at the approach of convalescence."

Our author cautions the reader against regarding these symptoms as arising solely from disturbance of the mental faculties, and requiring only moral remedies; and then proceeds to protest against the idea that "physicians particularly conversant with, and eminent in the cure of, the disorders of the mind, are not to be entrusted with those that ordinarily and exclusively happen to the body." (p. 55.) This idea Dr. Willis holds to be founded in misconception, and thinks, on the contrary, that "his acquaintance, by means of his mental practice, with almost every variety of disorder to which the body is liable, renders him surely competent and able to prescribe to the complaints incident to both body and mind, be they of whatever kind or character they may." (p. 56.) We suspect, however, that our author will find it a hard task to persuade any one that the physician, whose time is occupied in studying one complaint, thereby acquires a knowledge of another bearing no analogy to it whatever, or that the man, who is most skilful in soothing a paroxysm of madness, becomes, by this "mental practice," equally expert in relieving a paroxysm of the gout.

From this short digression, we return to the bodily symptoms which accompany the high state of mental derangement. These are frequently sufficiently obvious; but we think Dr. Willis has scarcely been judicious in some parts of his enumeration, particularly where we are informed that the patient "resists all advice, as well as control, more especially if it be attempted by any of his own family or servants." (p. 57.) The first symptom properly belonging to this class is the expression of the countenance: the face is generally more or less bloated, and the upper eyelid raised so as to expose the conjunctiva above the iris, giving a wildness to the eye that may, to a certain extent, be imitated artificially; the pupils are dilated, and the eyelids in constant motion. Some degree of head-ache is frequently complained of, or else there is an "unusual sensation" in the head. The pulse is increased in frequency, and the tongue is white and tremulous, sometimes becoming furred and brown, or even black. Salivation is rather a frequent occurrence. The heat of the body varies; the skin is sometimes dry, and sometimes covered with partial perspirations. Anxiety is often experienced in the precordia, and the breathing becomes hurried and irregular. The stomach is insensible to emetics, and the bowels not acted upon by the usual doses of purgatives; blisters produce little pain and scanty discharge; the evacuations pass insensibly: in short, every organ will be found more or less disordered, and the natural sense of feeling throughout the frame perverted." (p. 60.)

In the third chapter, the author treats of the remote and proximate causes. The remote causes are divided into those which act primarily on the body, and those which act primarily upon the mind: the effect of spirituous liquors illustrates the former, and the effect produced by his daughter's ill-treatment on the mind of Lear is adduced as an example of the latter. Dr. Willis here enters into a medico-critical disquisition on this sublime tragedy, which occupies not less than eight pages. These we must crave permission to pass over; for, although we admit that Lear, as performed by KEMBLE, afforded the most interesting lesson on the subject of madness we ever received—at any theatre, yet we much fear our readers might suppose a Number of the "New Monthly" had been sent them by mistake, if we entered upon this discussion.

On the subject of the proximate cause, we, of course, gain no satisfactory information: although the author regards the nervous system as in fault, he denies the justice of conclusions drawn from examinations after death. In allusion to some descriptions of this kind, we have the following judicious observations:

"That those symptoms which are usually termed symptoms of a preternatural activity, or an increased action of the vessels of the brain, such as pain and sense of fullness in the head, flushed cheeks, suffused eyes, and dilated pupils, are mostly present in those cases where delirium or derangement occur, I readily admit; that, upon an examination after death, we find a turgescence of the blood-vessels, and much other deviation from the healthy state, I also admit; but that these, either separately or conjunctively, are the causes of the delirium, I should deem improbable. Does a hot skin, for example, produce a pain in the head?—a pain in the head, a thirst?—or a thirst, a quick pulse? All these bespeak a general affection of the constitution. Confusion of ideas, derangement, and delirium, are also symptoms which mark an increased disorder of the system.

"Does any one attribute the delirium of a drunken man to his quick pulse, his flushed cheeks, or glistening eyes? or imagine his death (knowing him to have drank an immoderate quantity of spirits,) to be caused by a turgescence of the vessels in his brain? Were we to starve a man to death, the brain must necessarily undergo some alteration; but would it be consistent in us to attribute his death to that alteration, when both the one and the other will admit of a much more rational explanation?

"Of these different opinions, I apprehend that of Dr. Cullen to be nearest the truth; which supposes that a peculiar state of the nervous system is the proximate cause. In proof of this opinion, I shall not go into the dissecting-room, and examine whether a brain is hard or soft, dry or moist, loaded with blood or otherwise; because, having ascertained this, I should then have to learn which of these conditions is best adapted to a sound mind; which of them is a cause, and which an effect, of derangement; and by what applications these states or conditions could be altered or improved.

"Necessary as it certainly is for a physician to examine the dead body, and to make himself acquainted with the anatomy of the human frame, how much more requisite is it for him to acquire a knowledge of the living body and of its disordered functions,—an attention to which can alone materially tend to any practical good. Dissection may lay open to his view many unhealthy appearances; but can it explain to him why ipecacuanha sickens, or aloes purge; why wine intoxicates, or death follows starvation? or can it assist him in preventing these effects? Such information can surely never be obtained from a dead body."

The author, regarding excessive irritability as constituting one of the leading features in this disease, reprobates in strong terms the use of any means calculated to debilitate the constitution. The principal form of depletion is blood-letting; and against this his remarks are more immediately directed, and the censure extended to delirium. "Whether the patient be old or young, strong or weak, whether labouring under the high or low state of this disorder, it is deemed a case for the lancet or for cupping, and (erroneously, I am persuaded,) blood is copiously drawn from the patient." (p. 90.)

Dr. Willis is of opinion that his "high state" corresponds to the phrenitis of the ancients, and the authority of various writers, from Hippocrates downwards, is appealed to in support of the position: we are not inclined to deny it. In examining the method of treatment, the patient is considered with regard, 1st, to the preservation of his life; 2d, his irritability; 3d, his general health; 4th, his mental disorder. The first of these certainly differs only in the form of expression from the indication given by CULLEN for the cure of fever,—viz. to obviate the tendency to death: we leave our readers to decide which of the two is the better. It must be acknowledged that, the more violent the delirium under which a patient labours, the greater is the danger to be apprehended; and, as the irritability of a man in his sound mind may be alleviated by remedies which procure rest and give support, so it is argued by Dr. Willis that it becomes much more necessary to employ the same class of remedies, when the irritability is increased to such an extent as to threaten life. With a view, and certainly with the effect of illustrating his opinion, the author relates the following case:

"The patient was a young lady, of a naturally irritable constitution; who, having been in a very nervous state for many months, was, from domestic occurrences, thrown into a most violent delirium: on the sixth day from the attack of which she was placed under my care. With short intervals of cessation, she had been continually raving for four successive days and nights; labouring, at the same time, under such irritability, that four persons had been employed to watch and prevent her from getting out of her bed. While in this state, and previously to her becoming my patient, leeches had been applied to her forehead and temples, cupping-glasses to the back of her neck, and a blister to her head; purgatives also were given. Barley-water, with weak broth, had been the only sustenance allowed. Her state, as I found it, was this: she had ceased to rave, probably from exhaustion, having been wholly without sleep; she had become obstinately silent, but was still in perpetual motion; her pulse was 130, her whole skin very hot, and completely parched; her face flushed and bloated; her eyes suffused with blood, and wide open, yet she could discern nothing; she was also unconscious of her evacuations; her tongue was brown; her lips and teeth covered with sordes. In attempting to feed her with a spoon, she clenched her teeth: if we succeeded in putting any thing into her mouth, she spit it out after keeping it there a moment; so that it was impossible to administer any medicine without using force. Had the lady died in this state, and dissection been desired, a turgescence of the vessels of the brain, water effused into its ventricles, or some other deviation from the healthy state, would probably have appeared. Her death might then have been attributed to one or more of these circumstances.

"Viewing this case differently, and considering that she had been incessantly raving, till, from exhaustion, she could rave no longer; that she had not closed her eyes for five successive days and nights; that weak broth had been the only sustenance allowed her, I inferred that, although there might be some disease in the brain, either congestion of blood or effusion of serum, the patient was necessarily nearly worn out, and her life in danger.

"Under this impression, therefore, I immediately ordered her two glasses of old port wine, and two hours afterwards three ounces of a decoction of bark with some of the tincture, as the only means of saving her life. In four hours from my first seeing her, she was in a sound sleep, but only for a short time. Upon her awaking, the same quantity of decoction of bark was again given, when she slept three hours together. On the following morning, her life was comparatively safe: although she was still unconscious where she was, and took no notice of persons in the room, she no longer clenched her teeth or spit; but, when breakfast was offered to her, she put the cup naturally to her mouth; and, after obtaining more sleep from a continuance of these remedies, she was able to answer questions correctly. In short, her irritability began to subside, and her sense of feeling to return in some degree from the moment they were first applied."

The result of this case sufficiently shows that the method of cure adopted by Dr. Willis was beneficial; and we give him all credit for the superior sagacity displayed by him on this occasion: but we feel convinced he will acknowledge, on mature reflection, that his subsequent reasoning is inadmissible, when he asks, "as in this instance a change from a lowering to a strengthening plan brought the patient" from a state of great danger into one of safety, is it not a fair conclusion that tonics, in cases of delirium, ought to be prescribed? "and that medicines which enervate the patient should be avoided?" Most assuredly, it is not a fair conclusion; nor by any means a safe one. The case above related proves (what every experienced physician must have met with,) that there are certain cases of delirium, with great irritability and exhaustion, requiring stimulant remedies; but it does not by any means show that all cases are of this nature, nor that the majority are not totally different in their causes and method of cure.

The *irritability* of the patient next comes under discussion; and this is evidently the point on which the whole of the author's pathology rests. The same medicines, we are informed, which are calculated to preserve the life of the patient from the effects of delirium, are equally calculated, when given in smaller quantity, to prevent him from falling into that state; and therefore wine, bark, musk, henbane, hemlock, tartar emetic, and foxglove, "may, all or each of them," be administered to allay the irritability of either state. We speak with deference; but what species of irritability may that be, in which wine, bark, and musk are indicated, and the most opposite medicines in the materia medica—tartarized antimony and digitalis, likewise proper? Cinchona is regarded as a remedy of great efficacy in irritability proceeding from debility, the result of blood-letting, puerperal fever, or typhus: in such cases, the bowels must be kept open during its administration. "In proportion (says our author,) to the quickness of the pulse, and increase of heat and irritability, the more is this medicine requisite." (p. 124.) Conium and hyocyamus require some degree of caution in their exhibition; and the same remark applies to the tartar emetic: if a patient, who is in no danger, be so irritable as to talk constantly, a fourth of a grain might nauseate, and so quiet him; but, if this be repeated too frequently, he would be weakened, and thus the irritability increased. Opium is reprobated as injurious.

The *general health* comes to be considered when the immediate danger is over, and the irritability diminished. On this subject all that we find is, "that a combination of medicines capable of acting mildly upon the whole system" ought to be ordered two or three times a-day, with baths, exercise, &c.

When the bodily health is restored, the *mental disorder* still continuing, one of the first and most important indications is to procure sleep. This is best facilitated by the narcotics above mentioned, and exercise carried to such an extent as to produce moderate fatigue. Any attempt to divert the mind by the introduction of friends, "the exhibition of pictures, the representation of plays, or similar amusements," is regarded by our author as useless; but he thinks it reasonable to infer, that any thing capable of communicating a sudden shock to the body may produce a favourable impression on the mind; and asks, "what then is likely to be better calculated for the purpose than an emetic?" The opinions of Drs. Munro and Cox, and of Mr. Hill, agree with that of Dr. Willis; but that of Drs. Hallaran and Haslam is directly opposed to it. "I cannot (says the former writer,) too forcibly resist the practice of administering emetics to insane patients, in such doses as may suddenly promote violent action of the stomach, at a time when the vessels of the head may be surcharged with arterial blood, and when the danger of over-distention is to be apprehended." This point can only be determined by experience; and we have not ourselves had enough of this to venture an opinion. Blisters, applied at a distance from the head, are recommended as very serviceable; and this part of the subject terminated with the following observations:

"Although I have now considered the method of treatment under four heads, I do not mean that we are strictly to confine ourselves to the rules or advice contained therein, in every case indiscriminately. I do not contend, that wine and bark are always to be given in the first instance; that an emetic is never to be prescribed until the bodily health is restored; or that blood is, on no account whatever, to be taken away. There may be cases of this disorder in which blood-letting may be required, and prove serviceable; but such I believe to be very rare. To give wine and bark when the stomach and bowels are loaded and confined, would doubtless be injurious. Emetics and purgatives would at this time be the most necessary and useful remedies."

"My object, in making this division, has been rather for the purpose of laying down more clearly the principle upon which we should attempt the cure of a disordered mind; of explaining why we must not look for its restoration until much improvement has taken place in the health of the body; of pointing out the effects, both advantageous and injurious, that may happen to arise from our remedies, and of demonstrating that we must be directed in our practice, not by the dissection of the dead, but by an attention to the living,—by an examination of the symptoms, not only both past and present, but also of those which are to be apprehended from the countenance, behaviour, irritability, and disordered functions, both of the mind and body. By such symptoms must we be always guided in our practice; bearing in mind that patients, from the very nature of this disease, are, as we have before observed, likely to sink suddenly, unless supported. Where we think it necessary, therefore, to employ such means as have any tendency to weaken or lower the system, we should do it with caution; watching their effects both on the mind and body, lest a state of danger should surprise us when least expected."

The remaining part of this section is occupied with general remarks, and a biographical sketch of the author's uncle, Dr. Willis.

The next chapter consists in a description of the causes and cure of the *low state*, and its identity with the lethargus of the ancients. The subject, however interesting, has been so lately discussed in this Journal, when the work of Dr. Falret on Suicide was reviewed, that we decline entering upon it at present.

The work terminates with some cursory remarks on *lunacy*, from which we select the following passages, which, if not applied, are at least applicable, to a recent memorable trial.

"Were we introduced to the patient with a view of undertaking his cure, we ought certainly to be admitted into his presence with the very influence upon which we are hereafter to act; we ought to use no deceit, but openly and firmly, though delicately, acquaint him with his situation: but we must be careful how we confound this with a visit intended for the mere object of examining whether a person be sane or insane. For, knowing how much it must distress and alarm a man to be told he is deemed a lunatic, and considered incapable of managing himself and his affairs, we may expect the result of such a communication to be, either an attempt on his part to conceal what we wish to discover, or a state of agitation very difficult to be distinguished from a paroxysm of the disorder, and equally tending to mislead the judgment of the inquirer.

"The visit of the physician ought, therefore, to appear natural and undesigned, while its real object should be carefully kept from the knowledge of the patient; who, being speciously led to that conversation upon which his supposed derangement turns, will, if without suspicion, give immediate proofs of it, by voluntarily disclosing his delusions. Since, also, it is confessedly an act of humanity to pronounce a man insane who is *bonâ fide* such, so is it equally humane in the physician to ascertain the truth, if possible, without unnecessarily wounding the feelings of the individual.

"During the course of the inquiry, a question may arise as to the difference between a sound, a weak, and an unsound mind, or between an unsound mind and lunacy.

"In attempting an explanation of these different states, I shall wholly abstain from entering into theoretical, legal, or metaphysical definitions, and strictly confine myself to a consideration of them in a medical and practical point of view,—to a statement of facts, indeed, obtained from observation.

"A sound mind is one wholly free from delusion. Weak minds again only differ from strong ones in the extent and power of their faculties; but, unless they betray symptoms of delusion, their soundness cannot be questioned. An unsound mind, on the contrary, is marked by delusion, by an apparent insensibility to, or perversion of, those feelings which are peculiarly characteristic of our nature. Some lunatics, for instance, are callous to a just sense of affection, decency, or honour; they hate those, without a cause, who were formerly most dear to them; others take delight in cruelty; many are more or less offended at not receiving that attention to which their delusions persuade them they are entitled. Retention of memory, display of talents, enjoyment in amusing games, and an appearance of rationality on various subjects, are not inconsistent with unsoundness of mind: hence, sometimes, arises the difficulty of distinguishing between sanity and insanity."

---

Art. II.—*On the Principles of Inflammation and Fever.* By C. E. Lucas, m.d.—pp. 304. T. and G. Underwood, London, 1822.

So deeply impressed are we with the importance of sound principles in pathology, and so conscious of the imperfections even of the *modern* code, that we always take up with pleasure any work which undertakes to improve us in this department of medical science. Dr. Lucas, with great modesty, disclaims all intention of adding to the stock of our *facts.* He is content to take those which are already known; and his object is to point out, more fully than has hitherto been attempted, their various bearings and connexions,—to illustrate the general features of disease,—to direct the true course of future inquiries,—and to establish, if possible, rational and consistent views on subjects of great practical importance. Inflammation, and its many modifications, are the chief objects of the author's inquiry; and no one, we think, practically engaged in the profession of physic, but must acknowledge it to be of paramount interest. It is singular, certainly, that the opinions of medical men should still continue so unsettled on a point of such consequence; and that, in 1822, it should be deemed adviseable to enter into a full investigation of the doctrine of inflammation. Of the propriety of such an attempt, we needed no other monitor than our own conscience to convince us; and if any of our readers, in the pride of their hearts, should question this, we think they would be converted by a candid examination of these pages.

Dr. Lucas has acquitted himself of his task in an able manner. He is too good a pathologist to suppose that he has broken down all the barriers of difficulty, and left nothing to posterity but acquiescence and admiration; but he may have done a great deal, and be still very wide of this consummation so devoutly to be wished. He possesses many requisites for the undertaking he engages in: a practical acquaintance with his profession,—very clear notions of general pathology,—a *copia verborum*, which few can hope to attain,—and a very honourable desire to do justice to the merits of preceding authors. We rose from the perusal of the volume, indeed, very favourably impressed with the intelligence and industry of its author; and we conscientiously recommend it to the notice of our readers. That it has faults, (and what work, upon far less intricate topics, has not,) it would be in vain to deny; but they are light when set in the balance against its various merits.

In justice to ourselves and to the author, we shall now enter upon a detailed examination of the work; again reminding the reader, however, that, as it makes no pretensions to *novelty of matter*, so his expectations must be limited to finding what musicians call *variations* of an old tune, or what the world calls an old friend with a new face.

The author begins by offering some observations on the nervous system, explanatory of its influence in the production of disease; and he then proceeds to inquire into the action of blood-vessels, more particularly of the capillaries, and their relation to the central organ of the circulation. The object is to show the different causes of *effusion* from the exhalants, all equally depending on the relation of the momentum of the blood to the resistance offered in the extreme vessels. They are, increased action of the heart with plethora of the arteries, obstruction of the veins, and loss of tone in the exhalants themselves. The consideration of the action of vessels in their ordinary state, leads to that of their action under inordinate excitement, and therefore, at page 45, the author plunges into his subject. He divides it into two chapters, the first of which treats of the proximate cause and modifications of inflammation; the second, of the remedies for inflammation. We think the work would have been much improved by a further subdivision. If the author has ever travelled, he cannot have failed to perceive the tedium of a twenty-mile stage: akin to this is a chapter of 103 pages, in a volume which contains but 300 in all.

Dr. Lucas is a supporter of that theory of inflammation which, whatever may have been its origin, has chiefly risen into notice by the experiments and reasonings of Dr. Wilson Philip and Dr. Hastings. He defines inflammation to consist in an *impeded or obstructed state of the capillaries, with increased action of the contiguous vessels;* and he is at great pains to combat the old notion of its being *increased action* of vessels. Now, on this subject of the *essence* or intimate nature of inflammation, we have just one observation to make. Dr. Hastings finds dilated vessels, and a languid circulation, in the inflamed web of a frog. We are ready to acknowledge this is an important fact; but we object to the terms *debility* and *weakened action* of the capillaries, as applied to this state of *over-distention.* All parties are agreed that there is increased action somewhere. John Hunter said it was in the *inflamed vessels* themselves. Dr. Philip says it is in the *larger arteries;* and Dr. Lucas, in the *contiguous vessels.* All are agreed, too, that there is a disturbance of equilibrium between the action of different sets of vessels; and in this, inflammation appears mainly to consist. That there is no essential distinction between the two theories, must be obvious from the fact that the remedies for inflammation recommended by the partizans of each are the same. The more correct explanation of the phenomena we believe to be that of Dr. Hastings; but much, very much, is wanting before we shall be able to define accurately in what consist the differences between simple increased action of vessels, or irritation, congestion, and actual inflammation. The subject appears to us, from its very nature, to be beyond the presumable limits of human investigation, and we cannot but express our surprise that so much importance has been attached to it.

The author goes on to notice the *consequences* of inflammation, and he expresses himself in the following clear manner:

"In a disorder combining congestion with increased action of vessels, it is obvious that there will be a natural tendency to relief by effusion. This, which has been already mentioned as the constant accompaniment of inflammation, will be modified by the stage and degree of the disorder, and the nature of the parts affected. The effusion, therefore, will consist either of such fluids as the vessels are naturally destined to throw out by secretion, of some unnatural or modified secretion, or of the blood itself; so nearly are hemorrhage and dropsy connected with inflammation. Thus the skin, and serous membranes, will most readily pour out serum; the mucous membranes, mucus; and the different glands their respective secretions generally, mixed with other fluid matters, giving them a more anomalous character." (p. 56, 57.)

"But there are other effusions peculiar to inflammation. The coagulating lymph of the blood may be mentioned as one of these, being separated from the other constituent parts of the circulating fluid, and thrown out of the vessels; and pus, a fluid distinct from all others in the animal body, is a frequent product of inflammation. These we shall here briefly notice.

"It appears that, whenever the vessels act with unusual force, there is a tendency in the coagulating lymph to separate from the other constituent parts of the blood. This not only shows itself by the effusion of this lymph in active inflammation, (hence called by Mr. Hunter, adhesive inflammation,) but is seen in the blood itself drawn during inflammation, or any state of general increased action of the vascular system, by separating from the general mass, and coagulating firmly on the surface. This has generally been ascribed to the slower coagulation of the blood in inflammation, thereby giving more time for the spontaneous separation of that fluid into its constituent parts; but it has always appeared to me to depend rather upon a looser combination of those parts, as the separation is very distinctly visible long before the coagulation of the fluid, which I have also not observed to take place sooner than that of healthy blood. I therefore conceive that the inordinate action of the vessels in inflammation tends to separate the coagulating lymph from the other constituent parts of the blood during the circulation, in the same way, though not to the same degree, that agitation of healthy blood, when removed from the vessels, by stirring or otherwise, will do, causing this lymph to accumulate around the body putting the blood in motion. Whether this arises from the increase of heat generated by friction, or otherwise, the fact is, I think, sufficiently manifest. Accordingly, we find that it is under the most active condition of the vessels in inflammation that this coagulating lymph is thrown out, by the effusion of which, as the most tenacious part of the blood, it is probable that the circulation of the remaining part is facilitated, independent of the relief obtained by the diminution of volume." (p. 59—61.)

The observations which follow on *suppuration* and *gangrene* are conceived in the same spirit, and expressed with equal elegance. There is much truth, and we suspect also some novelty, in the succeeding remarks on spontaneous gangrene.

"In external injuries, mortification must, of course, frequently happen from destruction of vessels. But it also occasionally takes place without previous inflammation, apparently from loss of power to propel the blood to the extremities. It is wonderful, in some of these cases, where a great degree of torpor pervades the system, how long the living parts will remain united with the dead, without any attempt at separation; whilst in others, where this torpor does not prevail, we often witness the most astonishing efforts to remove the mortified parts by long continued and most extensive ulceration.

"It is not uncommon to find old men with large eschars on the forepart of the legs, formed by their sitting so close to the fire in cold weather as to be actually burnt by it. These dead parts frequently remain without producing any attempt at separation on the part of the living attached to them. But the affection I allude to above is the complete extinction of the life of the extremities in some cases, from cessation of the circulation. In a woman under forty, whose constitution had been undermined by intemperance, the fingers of both hands died in this way nearly two years before her death, without the least attempt at separation." (p. 66.)

The *strength of action* of the vessels in inflammation has obviously an important bearing on the course and character of the disorder, and consequently on the treatment. The author therefore next enters upon the investigation of the circumstances which *modify* the progress of inflammation. He notices, first, the influence of season, mind, and whatever can affect the general vigour of the *system*. He alludes, secondly, to the modifications arising from difference in the structure of the part affected. In reference to a question lately agitated, regarding supposed differences in the *nature* or *act* of inflammation, we find him thus expressing himself:

"We may, therefore, conclude that inflammation is in every instance essentially the same in its nature, though its character will vary considerably from the circumstances above mentioned; a truth which is daily enforcing itself upon our attention, by the advantages derived in the treatment of venereal and gouty inflammation, by directing our chief care to the degree of inflammation, rather than to its specific character; as well as by the disastrous results so frequently observed to follow the contrary practice of neglecting the treatment of the local disorder upon the acknowledged principles of inflammation."

The third circumstance which Dr. Lucas brings forward, as leading to modifications in the phenomena of inflammation, is one which has received but little attention from modern pathologists: we mean *morbid changes of the fluids* of the body. The revolutions of fashion may, perhaps, bring back again hoops and high head-dresses; but few of us, probably, ever anticipated a return to the doctrines of the humoral pathology. "It has been overlooked," says Dr. Lucas, (p. 70,) in the complacent satisfaction with which we regard the action of the solids, in preparing, perfecting, and preserving the circulating fluid, as the pabulum vitæ, by the different functions of digestion, assimulation, and excretion, that, if these important functions languish, the consequence will be, that the circulating mass must undergo a proportional morbid change. Doubtless, the proper remedy to be sought for is the restoration of the defective organs to their original integrity of function; but it is equally manifest that, in the mean time, the depravation of the fluids must bear its full share in the disorder produced. In short, if the fluids are prepared by the action of the solids, their healthy state must depend on the integrity of function of the latter, and the general health of the system equally on that of both, as integrant parts of the whole; and we may rest assured that any views in pathology excluding so palpable a truth must be defective."

Among the varieties of inflammation modified by the presence of a morbid state of the fluids, Dr. Lucas notices the scorbutic and venereal; the eruptions of the exanthemata; perhaps also gout; and occasionally, though not necessarily, the scrofulous and erysipelatous inflammation. On each of these topics the author descants at length, and, from the frequent mention of this doctrine, it is clear that he attaches much importance to it. We are inclined to think that Dr. Lucas has, in a considerable degree, made good his point. His mode of treating this question in reference to gouty inflammation, will afford a fair specimen of his general style of reasoning.

"It may, indeed, be doubted whether there is in gout any evidence of a morbid state of the fluids, as necessary to its production; the active symptoms of inflammation not evincing its presence by any modification of external character: but it appears to me, that the peculiar acid smell of the perspiration from the part affected, and the chalk-like depositions to which the disorder gives rise,* may be taken in proof of its existence. The great length of time, also, to which the disorder is frequently protracted, and that by repeated paroxysms in different and distant parts of the body, often to the length of many months, where there would seem no reason for the renewal of inflammation, affords much reason to believe that some peculiar acrimony is afloat in the system, operating as a morbid stimulus in the production of this specific inflammation. I am much strengthened in this opinion by the effects of the Eau médicinale, and other gout-medicines of the day, in procuring summary relief in the first instance, at the expense of more frequent visits of the disorder, till at length it is constantly present, and in some form or other proves fatal. The inflammation here is probably cured before the morbid matter can be thrown off, which therefore shortly renews its attack, while the powers of the constitution gradually give way under this unsuccessful conflict;† for it does not appear how the cure of inflammation, abstractedly considered, can be too rapid, if effected with safety to the organization.

"That there is something peculiar in the formation of gout, is universally admitted; and, when we reflect on the constant and intimate connexion it has with disorder of the digestive functions, I can see no reason why that peculiarity may not depend on the presence of some morbid

---

* "The gouty concretions, called chalk-stones, are found to consist of the urate of soda, the uric or lithic acid of which is, in a state of health, only to be found in the urine. Does not, therefore, this unnatural deposit by parts affected with the inflammation of gout, prove that some unusual proportion of its component principles existing in the blood is intimately connected with the formation of this disease, whether we suppose this to arise from some faulty action of the assimilating organs or those of excretion?

† "Whether the explanation here offered be correct or not, the fact of the use of these medicines being followed by more frequent attacks of the disorder, will not now, I believe, be disputed; the rage for their use which prevailed some time ago, and which is now happily gone by, having afforded ample and fatal experience of this truth, several instances of which have fallen under the observation of the author."

principle; especially when we find that this disorder frequently alternates with another of the urinary secretion, the principal resource of nature for the correction of the evils of a faulty assimilation."

Rheumatic inflammation, according to our author, presents no peculiarity of character, that may not be explained by the peculiar structure of the parts affected. Independent of those more commonly described, (such as membranous and ligamentous expansions,) the neurilema, or membranous condensation of cellular substance covering a nerve, obtains particular discussion; and *sciatica* is noticed as a well-marked instance of this kind of inflammation. The author's remarks (p. 85,) on the mode of treatment to be pursued in this and similar affections of nerves and their appendages, are useful, and evidently founded on the sure basis of experience. We think he is by no means so happy in his attempt, at p. 87, to account for the occurrence of rheumatic inflammation in particular habits, in preference to an analogous affection of a mucous or a serous surface. That this is not determined by chance, but that there is some assignable cause for the peculiar seat of inflammation in all cases, we are firmly persuaded; but Dr. Lucas's notions do not strike us as at all calculated to elucidate this intricate, but very curious, question. Such of our readers as are fond of dry theory may study the passage, and judge between us. In the treatment of acute rheumatism, the author, after premising a full bleeding and clearing the bowels, has found the most decided benefit from the combination of calomel with antimonial powder, and a small proportion of opium, given at short intervals, so as to affect the mouth slightly, but speedily. He states that, under this management, the disease is less likely to be followed by inflammation of the pericardium, than where the cure is entrusted either to repeated bleedings or to the bark alone. We think this hint very worthy of serious consideration.

The following remarks usher in the author's views of the nature and treatment of scrofulous inflammation:

"Scrofulous inflammation can only be considered as inflammation occurring in a habit where debility and irritability prevail constitutionally. It is obvious that, from the facility with which the balance of the circulation will be thus broken in upon, the scrofulous constitution will be particularly obnoxious to inflammation, which will generally show itself under the subacute form, and almost from the commencement display the character of weak and languid action. Acute inflammation will indeed often make its attack, yet it will not long preserve that character, but speedily fall into the same state of languid action with which it is more frequently marked from the beginning." (p. 98.)

"This view of the subject will readily suggest, for the general treatment of scrofula, the active employment of every means of improving the tone of the system, and the necessity of studiously avoiding all unnecessary irritation of body and mind, without which all our efforts for the former will be nugatory; and, for the local treatment, the use of remedies more or less stimulating, in proportion to the languor of vascular action." (p. 100.)

The indiscriminate use of the cold bath, as a general tonic in scrofulous cases, is strongly reprobated; and a very useful caution is given as to the education of children with a strong scrofulous taint.

To understand the nature of *erysipelatous* inflammation, on which the opinions of the world have been so long divided, the author states it to be "necessary to have a clear view of the functions and economy of the skin, as giving expression to the external symptoms of the disorder; and to consider the connexion of these with the more general disorder of the system." (p. 106.)

The state of constitution most obnoxious to the disorder is "that of natural or acquired delicacy, and feebleness of constitution." Hence it is most common in females, and in habits enervated by want of exercise, intemperance, and bad diet; in short, whenever the system is in a weak and irritable state. But, as the author remarks, erysipelas is sometimes an active inflammation, tending to suppuration, and accompanied by a strong and full pulse. Here "bleeding will generally be required, as well as other active evacuations." We would strongly recommend all those who are fond of *arguing* on the question of erysipelas to read Dr. Lucas's work attentively,—to mark the close analogy pointed out between this and every other form of inflammatory action, and inwardly to digest what is there stated regarding the varieties of character which it assumes,—and we will hazard the conjecture that they will not again engage in so fruitless an employment of their time. Dr. Lucas states, with great clearness, the influence of the nervous system on the action of vessels; and shows how, in crowded hospitals, and other situations calculated to depress the energies of the system, this form of inflammation appears characterized, as it is in most cases, by local and constitutional debility.

From the consideration of erysipelas, the author passes on to that of the febrile eruptions; but we do not find any thing in this part of the inquiry sufficiently interesting to detain us.

Chapter iv. is, as we have stated, dedicated to the *remedies* for inflammation; and the first discussion is on the *modus operandi* and comparative merits of local and general blood-letting. The subject is, we think, placed, on the whole, in a very fair point of view, though the bias of the author is evidently in favour of *topical* bleeding. The cure of inflammation on the principles of *derivation* and *counter-irritation*, next comes under review; and the warm bath, blisters, issues, and setons, are separately considered. This leads to an inquiry into the *internal* remedies used against inflammation, occupying a large portion of the chapter. Purgatives, nauseants, antimonials, mercury (especially in the form of calomel), mineral waters, sedatives, and lead, are successively considered. The influence of cold is then noticed, as a very important remedy, and one, too, of almost universal application. The last object of inquiry is the use of *stimulants* in inflammatory disorders; more particularly of heat, the oil of turpentine, cordials, and bark. Under the same head, however, are included additional remarks on the agency of purgatives, calomel, and cold; and we are further favoured with observations on the treatment of diarrhœa, dyspepsia, cholera, &c. This part of the subject is confusedly put together, and we think would be greatly improved by revision. Many of the suggestions thrown out are useful, and at the same time creditable to the author, but they are certainly not in their right place.

Having thus given a general outline of the manner in which the author has disposed of this portion of his subject, we shall lay before our readers some specimens of the mode in which he has executed his task.

Dr. Lucas is loud in the praises of tartar emetic as a remedy in the active inflammation of important organs. "This medicine (he says,) will be found of singular efficacy in inflammatory affections of the cerebral organs, particularly in young subjects, or in the middle stages of life. It will, indeed, be often necessary, in the more acutely inflammatory attacks, to premise bleeding, both general and local; but the greatest advantage will be afterwards obtained by the continued action of this remedy on the stomach; and in many cases, where the drawing blood might be objectionable from the general debility of the subject, it may altogether supersede the necessity for that evacuation." (p. 150, 151.)

In justice to the author, we think it right to add that, in p. 154, we find the following qualifying clause: "It is, however, incumbent on me to state that, when thus administered, it will require to be strictly watched; as it has occurred to me to witness its being followed, in one instance, by an inflammatory affection of the mucous membrane of the œsophagus and alimentary canal; which, with little intermission, dragged on for many months, terminating only with the life of the patient. Full doses of this medicine are also unsafe for infants, for which reason ipecacuanha should generally be preferred as an emetic for them."

We cannot avoid entering our protest against this severe discipline, watch it as strictly as we may. We are quite satisfied that a repetition of the bleeding would be infinitely less prejudicial to the system, and much more efficacious in the treatment of the disease. We are compelled, too, to notice the extravagant terms in which the author expresses himself of the benefit to be derived from calomel in the relief of internal inflammations. (p. 161 and 162.)

In our own practice, we have never found the necessity for such a plan of treatment; and we shrewdly suspect, if the author will omit it in the next simple case of pneumonia that comes under his care, trusting entirely to the lancet and antimonials, he will not have reason to repent the change.

Of the powers of calomel in the cure of croup, Dr. Lucas thus speaks:

"For many years past I have been in the habit of confiding the cure of this formidable disease to this remedy alone, for which it may be considered quite a specific. For this purpose it should be given in full doses, at intervals of one or two hours, till the symptoms give way, when it should be immediately laid aside, as it is not necessary to continue it with the view of bringing the system generally under its influence, and a little castor-oil given, with suitable mild nutritive diet, drawn from the vegetable mucilages and milk. In the most violent attack of this disease I ever saw, the patient, a delicate boy of four years, took 120 grains of calomel in little more than twenty-four hours, with complete success. In these cases, the disease is probably counteracted by the counter-irritation effected by the powerful action of the remedy on another and distant part of similar structure,—the mucous membrane of the bowels; the only sensible operation of the medicine being the bringing away dark-green membraneous stools, resembling boiled spinach, the breathing being relieved upon their appearance." (p. 163.)

The author is no friend to the employment of sedatives in the treatment of inflammatory diseases. We are perfectly disposed to coincide with him in his distrust of digitalis; though we think he greatly over-rates the dangers to be apprehended from the operation of the drug.

"As there is too much reason to believe that these medicines [digitalis and tobacco] have occasionally proved fatal, they should never be exhibited but with the greatest caution. Experience has now shown that, in phthisis, the excitement of the vascular system cannot be controlled with any degree of certainty by the action of digitalis; and, in a disorder inducing such dreadful debility, if not signally useful, it can hardly fail to be mischievous, unless given with the greatest caution, and in conjunction with other means counteracting its lowering effect. In inflammations it will be found perfectly nugatory in any thing like safe doses, until after free depletion, when its services may generally be dispensed with. In dropsy, particularly hydrothorax, though often bringing signal relief, it seems, on the other hand, to have frequently precipitated the fatal event. In conjunction, however, with other remedies, it may often render essential service." (p. 175.)

An exception is made in favour of hyoscyamus; and the author adds, "there is reason to expect that Dr. Granville's prussic acid will prove a valuable remedy." It does not appear, however, that Dr. Lucas has tried it in any active inflammations.

The reader will hardly wonder at the little faith which Dr. Lucas places in sedatives, when he comes to learn the importance attached by him to the use of stimulants in inflammatory diseases. This may readily be traced to the theoretical views of the nature of inflammation which he supports; and nothing can afford a stronger proof of the real influence which theory always must, in spite of ourselves, exert over our practice. If there is any one doctrinal point on which practitioners are agreed, it is on the mode in which purgatives operate in the relief of inflammations. Any gentleman, who has subjected himself to the effects of a little jalap and calomel for half a day, will be apt to subscribe to the notion of their *lowering* or *depressing* power;—but see how the theory of obstruction and debility can warp the judgment. "However we may overlook (says Dr. Lucas,) the *stimulant* property of these medicines in contemplating their action as evacuants, there can be little doubt that it is to this principally we are indebted for the relief obtained," (p. 196;) and again—

"With regard to the evacuation of serous and mucous fluids from the excretory vessels, and mucous follicles of the lining membrane of the bowels, it is usually too inconsiderable to have any great effect in reducing the volume of circulating fluids, and therefore to be of much importance in thus relieving inflammation; although it will doubtless assist in this respect as far as it goes, and it may be very important in diluting and carrying off any morbid acrimonious matter from the bowels. But the great advantage will arise from the *increased action produced upon a very extensive organ,* having the *most extensive and important sympathies, particularly with the capillaries of the surface.* Thus, it is probable that it is more by the new determination produced, than by their action as simple evacuants, that purgatives relieve inflammation; unless in those cases where the contents of the primæ viæ have undergone morbid changes, converting them into new forms of disorder." (p. 197, 198.)

The over-weening fondness of the author for his favourite theory (and consequently for stimulant remedies) in inflammation, peeps out on a thousand little occasions. Any author who happens to have advocated the employment of some such medicine, is sure to find favour in Dr. Lucas's eyes. Thus, at p. 96, the world is said to be greatly indebted to Dr. Balfour for inventing that most delectable mode of curing chronic rheumatism by rubbing, squeezing, beating, and kneading.

At page 112, the American mode of curing erysipelas, by smearing the inflamed surface with mercurial ointment, is talked of as being successful, and as affording (in conjunction with Dr. Balfour,) conclusive proof that the congested state of the vessels in inflammation depends on *loss of power*. Now we have pretty good reason to believe that, if the truth of this theory is to depend on the *success* of this trans-Atlantic practice, the author may bid adieu to it. It has been fairly tried at St. George's Hospital, and *found wanting*. But this is not all:—at page 202, the oil of turpentine, first introduced by Dr. Brennan, is extolled as a powerful remedy against puerperal fever; and the author even extends its use to peritoneal inflammation, not following the puerperal state.

It is curious to see how the author, between pages 209 and 214, (when treating of the inflammation of mucous surfaces,) vacillates between the employment of lowering and stimulating remedies; the former of which he is led to support from experience, and the latter from theory. The attempt made in pages 212 and 213, to establish a distinction between the mucous membranes of the stomach and bowels, so as to authorise the freer employment of stimulant remedies where the latter is affected, than would be safe in a diseased state of the former, we consider quite unworthy of so good a pathologist as Dr. Lucas has shown himself to be in the preceding chapter. The following sentiment, however, we perfectly coincide in:—"In affections of the stomach, it has appeared to me that inflammation is not in general sufficiently apprehended, and that stimulation is, in consequence, too indiscriminately employed in their treatment; the disorders of digestion ascribed to weakness of the stomach frequently depending on a subinflammatory state of that organ."

Some very useful reflections follow on the treatment of subacute inflammation of the stomach, forming the common dyspepsia of drunkards; but they are too long to quote. The reader will find them between pages 216 and 219.

The author is rather brief, we think, in his notice of the effect of bark upon chronic rheumatism. He merely states that "it acts by assisting the debilitated vessels in the recovery of their contractile powers, by virtue of its astringent principle; thus affording a further corroboration of the view here taken of inflammation, as founded in obstruction of the circulation from loss of power of the vessels." (p. 227.)

Here endeth Dr. Lucas's fourth chapter, and with it his Essay on Inflammation. The remaining pages of the volume are occupied with observations on Fever; or, rather, as he might have termed them, praises of Dr. Armstrong,—for really we can find little else in this part of the work. How this author will relish the admiration lavished upon his notion of the *typhoid principle*, (pages 276, 286, &c.) we cannot pretend to determine.

Probably Dr. Lucas is not aware that Dr. Armstrong's notions concerning the *origin* of typhus have lately undergone a great change. "Being now fully persuaded, (says Dr. A.) that I formerly committed an error in supposing human contagion to be the primary source of typhous fever, it becomes a duty in me to acknowledge that error without reservation."*

We have already had occasion to notice the too-easy credulity of Dr. Lucas on certain points of practice, but we hardly expected to find him gravely assenting to a notion entertained by some practitioners in the east, that "a slight salivation by mercury, kept up for a few weeks after the bite of a rabid animal, invariably prevents the development of the disease;" characterising this as a *valuable discovery*, and drawing conclusions from it with reference to the primary action of contagion.

We have already, however, exceeded our usual limits, and, as it is always painful to us to find faults, shall say no more of this Essay on Fever, than that it is decidedly inferior to that on Inflammation.

We cannot part with our author, however, in this mood. We must beg him, in conclusion, to accept our thanks for the instruction he has afforded us. If we have taken the liberty to differ on certain occasions, he must understand that it has cost us some trouble to pick out the errors; and, conscious that many passages of much merit have been passed by unquoted, (though we hope not unregarded,) we again recommend the work to the attention of our readers.

---

Art. III.—*A Short Treatise on Operative Surgery, describing the principal Operations as they are practised in England and France; designed for the use of Students, in operating on the dead Body.* By Charles Averill, Surgeon, Cheltenham; Fellow of the Royal College of Surgeons, London.—pp. 172. Jackson, London, 1823.

The title-page of this little work sufficiently explains its object, and we feel much gratification in saying that the execution is very creditable to the author, who appears to have attended the practice of the French surgeons especially with great diligence; of whose qualifications as operators he thus speaks: and what he says is quite in unison with our own sentiments on the subject.

"If the surgeons of France retain, in any respect, the superiority they were formerly acknowledged to possess over those of this country, it is as operators only; which can alone be accounted for, by the attention paid in the French schools to the practice of operating on the dead. This single branch of the science seems to be less insisted on, than

* See *Medical Intelligencer* for 1822, vol. iii. p. 170.

might be wished or expected by most English students in the dissecting room. It is usually taught in London as collateral to the courses of anatomical lectures, few of which are devoted to the subject; while the practical knowledge is chiefly obtained from cases furnished by the hospitals. These are the only public opportunities of acquiring a qualification so essential to the professional character, so important to society." (pages iii. iv.)

Our author says, that he is not aware of the existence of any *concise* work, the sole object of which is to enable the student to practise surgical operations on the dead subject; and, with this limitation as to conciseness, we agree with him, although we must not forget that excellent work, the *Cours d'Operations* of old DIONIS, enriched with M. DE LA FAYE's notes, and which even still deserves to be studied. Mr. Averill concludes his introduction as follows:

"The operations which are requisite for the cure of strangulated hernia, for hydrocele, for diseases of the eye, for contractions of the œsophagus, urethra, and intestinum rectum, for polypus in the nose, and some others, are omitted, as they cannot, in general, be practised on the dead body: but, since the confidence of the surgeon in performing them chiefly depends on his anatomical knowledge, the necessary qualification is now justly enforced, and ably explained, by the teachers of anatomy in London." (pages vi. vii.)

It is obvious that we cannot afford space to quote largely from a work of this description; nor can we do much more than recommend it to the junior members of the profession, since no analysis that we could make could put our readers in possession of the numerous descriptions of operations contained in it. In mentioning the ligature of the carotid artery, Mr. Averill is mistaken in supposing that Sir ASTLEY COOPER first performed that operation: but this is a trifling error. We shall subjoin the methods of taking up the posterior tibial artery, in three different situations, since we have reason to believe that the situation of this vessel is not so generally understood as that of some others.

"*Posterior Tibial at the Ankle.*—The patient being placed with his face downwards, make an incision two inches long between the inner malleolus and tendo Achillis, but nearer the former; cut through the aponeurosis, and you find the artery nearly under the malleolus, having the tibial nerve rather behind and to its outside, and an accompanying vein on each side.

"*Posterior Tibial rather below the Middle of the Leg.*—A little below the middle of the leg, begin an incision on the inner edge of the gastrocnemius; continue it obliquely for three inches in the direction of that muscle, so as to separate it from those beneath; elevate it with the upper part of the tendo Achillis, and, on the first division of the muscle beneath, you find the artery, with the nerve rather behind and to its outer side, and an accompanying vein on each side.

"*Posterior Tibial high up in the Leg.*—Begin the incision below and between the condyles of the femur; continue it through the integuments four inches down the middle of the calf of the leg; cut through the aponeurosis and gastrocnemius externus nearly to the same extent, till you come to the internus, on the inner side of the outer head of which you find the artery, with the nerve situated anteriorly and to its outer side, and the vein rather before it." (pages 30, 31.)

Passing over several very important operations, which however afford but little novelty, we come to the operation for the wry neck; and we shall quote the method employed by M. Dupuytren, in a female ten years of age, whose head had been awry for three years, and who was operated upon at the Hotel Dieu early in January 1822.

"The patient reclining against an assistant, a puncture was made, with a straight narrow-bladed bistoury, through the integuments just on the inner border of the sternal extremity of the contracted muscle. The blade of the bistoury, being flatly opposed to the muscle, was pushed cautiously behind it, the point being directed forwards and outwards till it protruded just on the outer side of the clavicular border. The edge of the bistoury was then turned towards the muscle, and a sufficient quantity of its posterior fibres cut to allow of the head being placed erect: the instrument was then withdrawn.

"In this way the integuments escaped being divided, and a future scar was prevented; a very desirable object, the patient being a female.

"The cut edges of the muscle were kept asunder by depressing the clavicle, and inclining the head to the left side. The former was effected by binding the right hand firmly to the foot, the knee being bent; thus the clavicular fibres of the deltoid drew the bone downwards: the latter by a roller passed round the head, and under the left axilla.

"The patient was kept in bed; and at the end of thirteen days the punctures were healed, and she had free motion of the neck, though, from long-continued habit, she still turned her face to the left side. The bandages were re-applied, and the same bodily position maintained till the 21st of February, when they were finally taken away, and the patient pronounced cured; the head being but very slightly inclined to the right side, and having free motion in every direction." (pages 62, 63, 64.)

In describing the operation of castration, our author informs us that M. Lisfranc, after the first incision, recommends the tumor to be dissected from below upwards, which prevents the blood from collecting before the point of the knife in the cellular texture of the scrotum. It appears that M. Richerand, in performing this operation, ties the whole cord; a plan which our author does not seem to approve, but to which we do not think there is any reasonable objection.

We shall conclude our review with describing M. Lisfranc's method of performing amputation at the shoulder and hip-joints, as being perhaps new to most of our readers:

" *Amputation at the Shoulder-Joint.*—M. Lisfranc recommends the following method, which, if dexterously executed, is certainly the most expeditious: it, however, requires considerable practice to accomplish it skilfully.

" Supposing the left extremity is to be removed, the patient is placed on an elevated seat; one assistant pressing the artery above the clavicle on the first rib, whilst another draws the arm forwards. The operator, standing behind the patient, with a long-bladed catling, pierces the integuments on the inner edge of the latissimus dorsi muscle, opposite the middle of the axilla, and pushes it obliquely upwards and forwards, till its point strikes against the under surface of the acromion; then, by raising the handle of the knife, its point is lowered, and protruded just before the clavicle, at the part where it joins the acromion. He then, by cutting downwards and outwards, forms a flap from the superior and posterior part of the arm, including the whole breadth of the deltoid muscle, and a part of the latissimus dorsi. This being held back by the assistant, the joint is cut through by passing the knife between its articulatory surfaces from behind forwards; and a corresponding flap is formed by cutting downwards and outwards between the muscles and bone on the inner side of the arm. The vessels being tied, and the flaps placed in contact with each other, the operation is finished.

" In operating on the right side, the patient should be seated on a low chair, and the catling thrust from above downwards, introducing it just before the point where the clavicle is connected to the acromion, and raising the hand as it is thrust backwards and downwards, till it appears on the inner edge of the latissimus dorsi, when the flap is to be formed, and the operation continued as before.

" M. Richerand observes, 'by this method a dexterous operator can separate the arm from the trunk as quickly as an expert carver detaches the wing of a partridge.'" (pages 134—136.)

" *Amputation at the Hip-Joint.*—On the dead subject, I have seen M. Lisfranc perform it with amazing dexterity, executing it in less than ten seconds. He adopts the following process:

" The nates of the patient resting on the edge of the table, and the extremity being supported by an assistant, the operator draws a line an inch in length, from the anterior and superior spinous process of the ilium, straight down the thigh. From this point he marks another inwards towards the pubes, of half an inch, so as to form a right angle. On the inner extremity of the last, he places the point of a long-bladed catling, and pushes it perpendicularly downwards, till it strikes against the head of the femur; then, passing it on the outer side of the bone, he thrusts it onwards, till it protrudes at about an inch from the margin of the anus. He now cuts outwards for near an inch, in order to get clear of the great trochanter, and forms the external flap, four or five inches in length, by cutting down the limb between the muscles and bone. The femoral artery, which may now be seen, is to be compressed between the fingers and thumb of an assistant; while the operator thrusts the knife in and out, at the same points as before; but, carrying it on the inner side of the head of the bone, he forms a smaller flap on that side of the extremity. He then, with the point of his knife, cuts through the capsular ligament surrounding the head of the femur, dislocates the bone, and removes the limb by dividing the round ligament, and the remaining adhesions. The blood-vessels being secured, and the flaps approximated, the operation is concluded." (pages 158—160.)

---

### DIVISION II.

### FOREIGN.

Art. IV.—*Leçons sur les Epidémies et l'Hygiene publique, faites a la Faculté de Medecine de Strasbourg.* Par Fr. Emm. Foderé, Professeur a cette Faculté.—pp. 523. A Paris, chez Levrault, 1822.

Although we much doubt whether the best executed work that the present state of our knowledge of the laws of epidemic diseases could produce, would really be of so much importance to mankind as might at first be imagined by those who have not considered the subject very deeply, we intend to give as extended an analysis of the labours of M. Foderé as our limits will permit. He is already well and favourably known to the profession by former productions of the same kind; and our doubts of the utility of such a work by no means arise from any apprehension that the subject will lose any thing of its importance in his hands. The volume before us is only one-third part of a great work, of the origin of which our author gives us a short account in his preface. It appears that the lectures upon Epidemic Diseases at Strasbourg, which were interrupted by the retirement of M. Rochard, were, by a decree of the Royal Council of Public Instruction, united to the chair of Legal Medicine, of which our author being professor, he determined to treat of this subject at length, as he had formerly done that relative to Medical Jurisprudence. In the month of March, 1822, having made great progress in his undertaking, he published a prospectus, requesting the assistance of his medical brethren to enable him to print this work, which he considered as indispensable. Unfortunately, this appeal scarcely produced him one-fourth of the subscribers necessary to effect his purpose; and we must therefore forgive him a few cynical remarks and a little grumbling occasioned by this disappointment. The first volume is therefore printed chiefly at the author's expense, and will be followed by two others, which are ready for the press, and will be published provided encouragement is given to the present one.

M. Foderé, like all authors, declares he has no intention to praise his own book, but that, as he has no system to defend, he has sought for the truth wherever he could find it; praising and blaming without hatred or envy, and with freedom and impartiality. Our readers will be able to judge hereafter whether M. Foderê is really the impartial and unprejudiced person he believes himself to be, or whether all the varieties of the passion love (including self-love) are not equally blind.

The volume before us is divided into three principal parts, or sections, prefaced by some general remarks, which occupy forty-three pages. The first section contains seven chapters, extending to nearly one-half of the book; the second section has also seven chapters; and the third, which commences with the description of the epidemic diseases *seriatim*, consists of two chapters only, each disease occupying one chapter. The aphoristic form which is adopted throughout the work, renders the task of analysis still more difficult. In a work which is evidently intended to be a complete treatise on the subject which it professes to treat, we must, of course, expect to commence *par le commencement*.

"It will not be imagined (he says,) that the ancients were without a knowledge of public hygiene. Who would believe, that in the time of those who constructed the zodiac of Denderah, that observations were wanting as to what was necessary for the preservation of health? The twenty thousand cities of Egypt would be a sufficient reply to such an absurdity. Tyre, Sidon, and the old Cyrene, which for a long time commanded the respect of nations, were strong, as much by their sanatory laws as by the boldness and intelligence of their inhabitants. The chiefs of these societies, who knew the carelessness of the people, gave to their laws the seal of religion." (p. 2.)

Forests placed so as to intercept the exhalation of marshes,—the salubrity or insalubrity of different places, as denoted by the inspection of the entrails of victims,—divine honours rendered to the Nile and other rivers, are so many proofs adduced by our author of the good sense of the nations of antiquity. Nor does it appear that the savages of the New World were less instructed as to the general advantages or disadvantages of particular districts relative to health, as they pointed out to Cortes the insalubrity of Vera Cruz, and to the Portugueze that of Pernambuco. These simple elements of medical knowledge, however, have given rise, in later and more refined times, to so many systems and to so many controversies, that truth has entirely been lost sight of, and the noble science of medicine been exposed to unmerited ridicule and obloquy. Our author goes on to give a rapid sketch of the different sects that have succeeded each other in the career of error, until the seventeenth century produced a revolution in philosophy generally, and led the way, through careful observation and an attentive study of the operations of nature, to a safe and more rational practice.

Our author, after some sensible remarks on the spirit of system which has, and ever will, we fear, prevail among the professors of our art, turns towards the particular subject of his work, and enumerates the principal authors who have written upon epidemic diseases during the last three centuries, beginning with Mercurialis, and ending with those works which the recent visitations of the yellow fever in Europe have made so familiar to the profession. "Materials then are not wanting (says M. Foderé); but we make bold to say that what is really wanting is a good criticism, an accordance of opinions, a stop to the spirit of party and faction, a good choice among so many contradictory opinions with which ancient and modern medicine is encumbered, and the establishment of a distinction between what is only supposed or marvellous, and what is real." (p. 12.) In these sentiments we entirely concur; but we do not see the necessity of reverting, upon all occasions, to the writings of Hippocrates and Galen to prove the truth of these assertions: we may come much nearer to our own time and country for an illustration, and we need only allude to the controversy regarding the Barcelona fever, to show that all the elements of a sound doctrine are as much a desideratum now as they ever were since the beginning of the world.

In recounting the various mistakes that have been made relative to the once favourite doctrine of the epidemic constitution of the air, M. Foderé mentions, in particular, the opinion of Sydenham with regard to the plague of London in 1665, and which that able physician, certainly very weakly, ascribed to the occult qualities of the air.

Our author, before he begins to state his doctrines, undertakes to root out some prejudices which have necessarily arisen from a belief in this unknown atmospheric influence; and, in the first place, remarks that the division of maladies affecting a population into epidemic, properly so called, and epidemic constitutions, and the refinements upon these distinctions, have been fraught with mischief, and from which some of the best modern writers have not been exempt. Our author instances the celebrated Frank, who believes that an epidemic has something particular in its nature, which, for instance, forbids the repetition of bleeding, or regulates the quantity of blood to be taken away, even in those diseases which are purely of an inflammatory nature. "Experience, however, proves (M. Foderé goes on to say,) that what is really inflammatory is always so, and requires an antiphlogistic treatment accordingly. It is true that in certain epidemics there is a mere appearance of inflammation, which does not always forbid bleeding, but which occasionally does so: we shall see, however, that this appearance is the effect of a principle analogous to that which produces malignant fevers *(fievres pernicieuses,)* and those diseases mentioned in our fourth section, and which consequently requires a treatment different from pure inflammation: but this principle is sufficiently known, and has nothing in common with the abstruse elements of the compilers of the doctrine of epidemics." (p. 19.) We shall merely remark upon this passage, that, from whatever cause it arises, we do not pretend to determine, but that nothing can be more certain than the very peculiar and marked character which diseases assume at some seasons, more than at the corresponding seasons of former years; and that the practitioner, whether he believe that such peculiarities are dependant upon atmospheric phenomena or not, must adopt and modify his practice to the circumstances of the time. The past winter in London affords a very fair illustration of the truth of this remark; and few practitioners, we conceive, will deny that the inflammatory affections that have been so prevalent have not demanded a different mode of treatment to that usually adopted, and that evacuations (particularly by the lancet) have not been so eminently and promptly successful in subduing the affection as has been generally found to be the case.

The second prejudice mentioned by M. Foderé is, in fact, the offspring of the first: it is, that an epidemic constitution puts an end to all other maladies, and which do not appear again until the epidemic has entirely ceased: to this we can have no objection. The belief in the possibility of contracting the germ of a disease in one season, and which shall only make its appearance in a succeeding one, is now so generally exploded, that we may be permitted to pass on to the fourth and last *prejudice* mentioned by our author, and which refers all diseases to some sudden change or instability of the weather. This leads him to consider the little assistance which our art has derived from the study of meteorology; for, although valetudinarians and delicate people are sensible of these influences, they have absolutely no power over the bulk of mankind, or, at best, can only be said to predispose these patients to some particular disease.

We come now to the definition of an epidemic, which is described to be a "certain malady very much diffused in consequence of one or more common causes, and of a predisposition to contract it, with which a number of individuals are furnished." The author before us evidently dwells more upon this latter circumstance (the predisposition of the patient,) than has usually been the case; and it forms, indeed, the principal feature of his opinion, and which appears to be much confirmed by observing the infrequency of these visitations now, compared with the preceding ages. "It is evident (says M. Foderé,) that the condition of humanity is ameliorated; for, since the system of the universe is not changed, and the atmosphere remains the same as in the first epoch after the creation, it is evident that there is nothing in the air above the scope of the human understanding or power. That which has been removed is filth, contagion, unwholesome habitations, bad food, and poisonous beverage. All this is true; but might not the cultivation of the earth, the clearing of forests, the draining of marshes, have also been dwelt upon, or at least enumerated; for, though the atmospheric air may be every where nearly the same, still its impregnations have been, and continue to be, frequent and fatal causes of epidemics, unless we are very widely mistaken. If there be difficulty, however, in searching into the essential causes of epidemics, the mode of treatment has been rendered not less difficult by the various systems and theories that have succeeded each other with such rapidity. To some of these M. Foderé alludes. PRINGLE, in consequence of certain experiments, introduced the practice of antiseptics. STOLL, believing every disease to arise from an epidemic bilious constitution, advocated the use of emetics in all cases. Brownism let loose upon the world a host of stimulating and exciting medicines; and latterly every thing has been referred to irritation, and depletion has become the order of the day. In this uncertainty, well may our author exclaim, "Combien je plains le jeune medecin qui debate dans une épidemie, et comment je plains encore plus ses malades! Cependant l'on guerit et l'on meurt avec toutes ces methodes." (p. 35.)

Let us turn now to something more satisfactory, and enumerate those circumstances which our author declares to be essential to render the history of an epidemic perfect and complete.

First, is the topography of the place; comprehending, besides the nature of the soil, &c. the construction and situation of the houses, the number of inhabitants, their manner of living, &c.

2dly. The climate, state and temperature of the air, the direction of the winds; and, if you wish to be very scientific, says our author, the state of the barometer, hygrometer, and thermometer, may be added.

3dly. The nature of the reigning disease, with its leading symptoms, their mode of accession, progress, and decline: in short, all the particulars usually considered indispensible to be known on these occasions.

4thly. The natural cures, as well as those produced by the medical treatment first adopted; together with that plan of cure which is considered most judicious.

5thly. A description of the origin and causes of the disease, whether it is simply an epidemic, or contagious also.

6thly. The methods of health proper to bring the epidemic to a termination, to diminish the contagion, or to confine it within certain bounds, &c. &c.

This enumeration comprises, pretty exactly, the whole plan which our author intends to follow in treating his subject, which he divides into eight sections; the two first being consecrated to the causes of these diseases, and the last six to the description of the numerous individual maladies, the methodical division of which will be found at the end of the first section, and upon which we shall have occasion to make a few remarks.

The nature of the preliminary matter of this work has necessarily compelled us to bestow more space upon this part of our subject than we had originally intended; but we now hasten to the first chapter of the first section, and which is thus entitled, "*On the Knowledge of Places, with respect to their Salubrity or Insalubrity.*" The subjects comprehended under this head are numerous, including the nature of soils, the direction of winds, the situation of forests, mountains, rivers, and marshes: whether the soil is alluvial or not; the temperature of the air, its dryness or humidity: after which come the sources of disease consequent upon civilization, such as those arising from manufactures, from a crowded population, from slaughter-houses and other nuisances, especially from burying-grounds in the vicinity of large cities.

With regard to the nature of soils, we find two important facts insisted on,—the fatality of the banks of large rivers, and of all those lands formed by the detrition of large bodies of water, and which are fatally shown in the instances of New Orleans and Walcheren; and, secondly, the unhealthiness of argillaceous soils above all other, and which retains and decomposes the moisture which it imbibes. But a slight acquaintance with mineralogy, says our author, will be sufficient to enable us to judge of the nature of the soil, and which an observation of the plants which grow upon it, and of which some examples are given. It is from this source that the Oases of the Arabian desert are insalubrious, as well as the desert itself at particular periods, and the sand of which covers a bed of argillaceous earth. We need not dwell upon the unwholesomeness of the vicinity of stagnant waters, especially if containing decayed animal or vegetable matter. Neither do we find much to detain us respecting the influence of those noxious effluvia with which the atmosphere itself is sometimes loaded. Our author's remarks are generally judicious, but they are not new,—neither do they aspire to the merit of novelty: and the same remark applies to what he says relative to general heat, humidity, electrical phenomena, and general direction of the winds. He quotes with approbation an observation of Sir G. BLANE's, that the violent operation of marsh effluvia has a relation in general to the heat of the climate, and which are more fatal in Zealand than in England, and still more so at the Equator than in Zealand.

We pause at page 61, and quote the following passage:

"In general, it is of the highest importance to fix our attention upon the nature of the predominant winds of a country, and upon their mode of acting upon the principle of life, for this action cannot be dissimulated; and, consequently, the winds contribute materially to form the climate, considered in a medical point of view. *That disgust of life which has for so long a time been remarked among the English, is singularly favoured by the dark complexion* (ton rembruni) *of the atmosphere of their island, by the moisture and storms which are permanent; and that the number of suicides is greater at London during the blowing of the east wind, which is sufficiently common in England, according to the testimony of Lind.*"

We really feel mortified at having met with so silly a passage in a book which contains the sentiments of a public teacher at a great university. Does not M. Foderé know that it is proved, as clearly as arithmetic can prove any point, that the number of suicides in London is inferior annually to that of Paris?—and, if atmosphere has to do with the question, why is London selected? We maintain, without fear of contradiction, that suicides in England, throughout *the country* generally, are not particularly frequent: and is it not more consonant to common sense and sound philosophy to attribute their occurrence in large cities to those agitating passions which there find their full development,—to the despair of the unsuccessful votary of fortune, the recklessness of profligacy, and the despondency attending sudden reverses of fortune, so frequent and so fatal in a commercial community?

Much has been said of the influence of the sirocco, or hot winds, in the south of Europe: our own personal experience leads us to believe that their inconveniences have been exaggerated, or, at least, that they depend as much upon the indolent habits and the mode of living of the natives as upon the wind itself; and certainly such effects as are described by travellers at Naples, Cadiz, &c. we have not experienced ourselves, nor observed in our own countrymen.

It does not appear that a continued rain, if the temperature of the air is low, is at all hurtful to the health; but, if the heat be great under the circumstances of long continuance of wet weather, severe disease is universally the consequence. The history of all hot countries abounds with fatal proofs of this truth.

"The country in general may be healthy, (says our author,) but there will be a cause of unhealthiness in some particular spot, and which will have been the cradle of the disease: thus, on one occasion, a putrid epidemic fever began from a deserter hid in a cavern, and who died of gaol-fever, which he communicated to those who succoured him. On another occasion, in a dry and healthy district, a violent epidemic broke out, in consequence of a body having been buried in a church too near the surface." (p. 67.) This is followed by an account of an epidemic which broke out in the neighbourhood of Nice, in a church-yard placed on a rock, and the earth of which was not sufficiently deep to inter the bodies.

The remaining pages of this chapter we must pass over, although much of the matter is interesting; but we shall extract some rules which M. Foderé lays down as necessary to secure and preserve the body in those hot and unhealthy climates, the fountain and birth-place of marsh-fever: and, 1st, we are recommended to observe very strictly all the sanatory laws relative to church-yards, privies, slaughter-houses, manufactures from animal remains, hospitals, prisons, &c. 2dly. To protect ourselves as much as possible from all natural and artificial stagnant waters. 3dly. To oppose a barrier to winds that are unhealthy, and to open a free course to those which are found to be salutary; and, 4thly, To preserve to the inhabitants a strong and robust constitution, capable of resisting the causes of disease, or of repelling them by a salutary re-action. The means necessary to accomplish these desirable ends are easily to be comprehended; and the best commentary upon these rules generally, perhaps, is the reply made by the post-master of *Torre de tre Ponti*, in the Pontine marshes, to M. Ozanam, who was surprised at the perfect state of health which this man enjoyed in the midst of a situation the most deadly. "I have dwelt (said he,) in this place more than forty years, and I have never had the fever: the only precautions I take are never to go out after the sun has set, nor before it is sufficiently high in the horizon, and to light a fire in the evening; I feed well, and drink wine; and that is the whole of my secret." In many parts of Holland, and in Zealand particularly, the precautions adopted by this sagacious post-master are precisely such as are found to be most efficacious in guarding the natives from the attack of that formidable fever which always prevails in that climate about the months of September and October, and from which the British army in Walcheren suffered so much.

Chapter ii. *on Solid and Liquid Aliment as Causes of Disease.*—We shall take the liberty of passing over, almost without comment, the first twenty pages of this chapter: it is filled with discussions that relate principally to the agriculturist,—the different soils proper for the growth of corn, the diseases which this class of plants is subject to, and their influence upon the animal economy, are noticed; as well as the invariable invasion of disease when the dearth of grain reduces a population to the precarious and unwholesome substitute of roots and herbs, as was exemplified at Naples in 1764, and more recently at Marseilles in the years 1812 and 13. Of course, the singular effects of the ergot are mentioned. With respect to animal substances employed as food, M. Foderé observes, that many epidemics have been common to men and animals; and that typhus especially has frequently reigned amongst horned cattle and the human species at the same period. In the year 1793, he says that a febrile dysentery was prevalent among the troops at Entrevaux, and which disease coincided with the arrival of cattle much over-heated, and affected with a discharge of bloody urine: in this state they were killed for the use of the troops; the flesh was very red, and went speedily into putrefaction. In 1799, in a village near Nice, a man died of anthrax, and his family were also very ill, in consequence of having lived upon the flesh of their cow, which died of the epizooty that prevailed at that time. So many accidents of this nature occurred during the epidemic among the cattle in 1744, that the exposing the flesh of these animals for sale was condemned by a decree of the parliament of Paris, (p. 117.) Many similar examples are given, and many arguments brought forward, in support of the principles maintained by our author on this point, and which have been controverted by many celebrated men. It appears to us, that, without attaching implicit credit to all the histories of epidemics which we hear of as arising from eating the flesh of diseased animals, that there is sufficient evidence to justify the exclusion of such food, and to render the enforcement of sanatory laws to that effect essentially necessary to the safety of every community.

The following remarks with respect to the effects of certain kinds of water on the human constitution, appears to us to be interesting.

"Water, the specific gravity of which is lighter than ordinary, is frequently more hurtful to the health than hard water. Having remained for a long time in a marshy soil, or in one enriched by the remains of organic matter, especially over a clayey bed, such water is impregnated with these remains and with mephitic gases, which render it very light, and which, occupying the place of common air, render it warm and disagreeable to the taste, as well as to the stomach. The inhabitants of communes so situated have a cachectic exterior, are dull, and digest their food with difficulty. The cattle of these districts partake of the same imperfections, and are subject to the same diseases." (p. 129.)

M. Foderé adds, that in these situations, in the months of August and September, putrid, worm, and bilious fevers, are epidemic.

M. Foderé is of opinion that the tænia, and other species of worms, are the product of the water; and he instances certain parts of the department of Doubs, on the confines of Switzerland, where he has seen children of six years of age frequently affected with those diseases. Of all the hypotheses that have been invented to account for the presence of worms in the human subject, this which our author has adopted is certainly among the most probable; although the minuteness of the eggs of these insects, which are scarcely visible even by the assistance of a strong microscope, has given rise to the belief that they are to be met with in the circumambient air, and that they may even be transmitted from the mother to the child, through the medium of the circulation or the milk. At all events, it must be evident, in either instance, that a predisposition in the individual must be necessary for their development.

Although water alone would be sufficient for mankind where the nourishment is otherwise salutary, and the situation of the country healthy and dry, our author thinks that fermented liquors are universally necessary in northern climates, and where the atmosphere is moist: but upon this point, as well as every thing regarding the use or abuse of wine, he affords but scanty information.

We proceed now to examine the third chapter, *on the Seasons and Variations in the Atmosphere as Causes of Disease.*—We are now arrived at an order of causes, says M. Foderé, which has served as a pretext for the greater part of the popular maladies which have afflicted humanity from the time of Hippocrates. Without totally disbelieving in atmospheric influence, our author very justly ridicules the abuse of this doctrine as contained in the older writers; though, perhaps, he dwells too often upon a point which is not only indefensible, but is not, that we are aware of, defended at present by any modern sect. It must be remarked, that the feelings excited by different states of the atmosphere, or the direction of particular winds, are only to be met with in the instance of delicate, feeble, and valetudinary subjects. It is also quite incontrovertible, we think, that certain maladies do really belong to particular seasons: thus, some of the exanthemata are found to be epidemic annually, at or about one particular period;—that, in this climate at least, the intestinal canal is the seat of summer disease, whilst the spring and winter more especially abound with pulmonary complaints. We cannot but think that M. Foderé has ascribed to particular seasons some diseases without sufficient grounds. What has dropsy to do with autumn? for example; or piles, or retention of urine?

We shall conclude our extracts from this chapter by giving, the result of the meteorological register kept by M. Brandes, professor at Breslau, whose diurnal observations amount to

180,000, and of which 70,000 were made by himself.—1st. The greatest degree of cold throughout Europe falls in the first days of January. 2d. To this generally succeeds a regular rise in the temperature, until near the end of that month, when a second return of cold ensues, and which reaches its height about the 17th of February. 3d. After this the cold decreases, and returns again, first in the eastern parts of Europe, then in the west and south, until the 9th, 14th, or 15th of March.—4th. After this the heat of the atmosphere rapidly increases; then for a few days it becomes more moderate; and then, in the southern countries, it uniformly increases from the end of March to the last days of April. 5th. The heat advances rapidly towards the 10th of May; but the warmth of the weather seems to be less regular every where about the beginning of June. 6th. The maximum of heat comes on sooner in the north than in the south: it appears to acquire two maximums,—one in the last third of the month of July, and the other from the 11th to the 16th of August. This is the common season of storms and tempests. 7th. A rapid and continual diminution of temperature begins in the middle of August in northern countries; but, in the beginning of October, this is usually suspended, and a latter kind of summer comes on: these alternations of heat and cold occur from the last third of October to the same period of November. In December, the diminution of heat in the northern countries is progressive: in the south, it is less evident in the middle of the month, but increases towards the end.

Following the arrangement of our author, we now come to a long critical examination of the different epidemic constitutions of authors, occupying nearly fifty pages, and constituting the fourth chapter. The order in which he treats of these is, first, those epidemics depending upon the irregularity of the seasons; secondly, those called catarrhal; then the history of malignant, intermittent, remittent, or typhous fevers, which have, under different names, devastated Europe, and which are evidently connected with a *marsh* origin. The variety of the particular symptoms of these epidemics is great; sometimes accompanied with petechiæ, with miliary eruptions, or confined to certain classes of men.

The first observation relative to the constitution of particular seasons mentioned by our author, is the example of Modena, from the years 1689 to 1694, as detailed by Ramazzini. That author attributes the diseases that ensued in this interval, consisting of fevers, diarrhœas, and colics, solely to the irregularity and great humidity of the first three years, and to the bad harvests which resulted as a necessary consequence. Nevertheless, the heat of the country, and the quantity of stagnant water, cannot but have been the chief origin of the mischief which these unusually wet seasons produced. The next example adduced is that of Rome, in the years 1703, 4, and 5, in which earthquakes were very frequent in the Roman state, notwithstanding the seasons were not otherwise remarkable for any unusual variations in heat or cold, dryness or moisture; yet BAGLIVI assures us that, in the spring, ophthalmia and diseases of the skin were unusually frequent; intermittent fevers in the summer; and, in autumn, the small-pox, apoplexy, and sudden deaths, were of every-day occurrence. The above-named writer very judiciously ascribes the principal number of these maladies to the continued alarms of the inhabitants,—to the quantity of salt-fish and other indigestible aliment,—and especially to the long and rigorous fasts which the inhabitants of Rome imposed upon themselves, for the purpose of diverting the divine wrath.

We shall not multiply examples, there are but too many; but from these it appears that there is no reason to ascribe the whole evil of an epidemic to the influence of the atmosphere. Bad seasons will produce bad harvests,—poverty combined with dearth,—a hot sun raising the exhalations from a soil overcharged with moisture, especially if encumbered with animal and vegetable remains: these causes combined will give rise to many formidable diseases, the particular character and symptoms of which will depend upon a combination of a number of causes, too minute to attract the notice of the philosopher.

We find it extremely difficult to make any selection from our author's remarks upon those catarrhal epidemics which have usually in this country been called influenzas, and of which many examples are given. The years 1780, 1803, and the last winter, are the most remarkable of these. It does not appear to us that a warm and moist state of the air has been necessary to the production of this complaint in this island: on the contrary, it has rather been met with in seasons of rigor; not where the cold has been regular and permanent, but where the atmosphere has been liable to great and sudden changes. Upon the whole, this chapter, though filled with the fruits of much reading and research, does not afford us a sufficient number of data to enable us to form any conclusions likely to be of use to us under similar inflictions.

Chapter v. *on Infection and Contagion.*—This chapter begins as follows: " *Que l'auguste verité est difficile a trouver!* I seek for it in the palaces of kings, in the national tribunes, in the courts of justice, in academies, in the writings of philosophers, in the books of physicians. One would have hoped to have found it, at least, among this last class, whose studies are supposed to rest upon facts, and who, seeing human nature in all its nakedness, ought not to be occupied with a rivalry of places, honours, and of vain distinctions: alas! it does not appear to be made for them; and there is amongst our profession Guelphes and Ghebelins, Whigs and Tories, as well as among those who are busied only with politics or family interests." (p. 189.)—[By the bye, our author, like the rest of his countrymen, finds it totally impossible to spell any English word correctly: thus we have *wigs* for whigs, *Crisholm* for Chisholm, and *Raffler* for Raffles, the late respected governor of Ceylon.]—The medical world, continues M. Foderé, is divided into contagionists and non-contagionists, and it is necessary to be ranged on the one side or the other, in order to avoid the charge of imbecility, or from the fear of making both parties one's enemies.

This prelude almost immediately introduces us to the opinions of our author upon the subject, which are stated soundly enough, and with that sort of confidence which implies not only a thorough conviction of the truth of the doctrines he entertains, but something bordering pretty nearly upon contempt for those who maintain a contrary sentiment. We may guess at the impartiality of this gentleman from his allusions to the last visitation of the yellow-fever at Barcelona. He strongly advocates the salubrity of that part of the coast of Spain; he appears to have the firmest reliance upon the report made by the French commission, which we have adverted to and exposed in a former Number of this Journal; he quotes their authority as incontrovertible, little aware, we trust, of the ample contradictions that have been given to the principal part of their statements, and the absolute weakness,—nay, we may add, falsehood,—of most of the premises from which they drew their deductions. We had much rather suppose M. Foderé to be ignorant of these counter-statements, than be obliged to believe that he has purposely omitted to notice them. Our readers need not be under any apprehension of our so soon renewing a controversy which we entered into so fully in one of the latter months of the preceding year: we shall only make one or two remarks, that arise out of the dogmas of the author before us, and which we would fain hope that we are only induced by a love of impartiality to submit to the consideration of our readers:—we trust that this is our motive; but, after what we read of and witness every day, we dare scarcely venture to affirm it positively. We were rather startled to find M. Foderé reproaching MM. Deveze, Valentin, &c. for denying the contagion of yellow fever at New Orleans, and which, he (M. Foderé) says, is acknowledged by the physicians of that place. Now, so far from this being the fact, we know that quarantine has been abolished at New Orleans by the authority of government, at the recommendation of the medical faculty of that town; that the profession is nearly unanimous in agreeing that the yellow fever is there of local origin; and, what is still more extraordinary, our author himself points out New Orleans as exactly the situation where this kind of fever may be expected to be found.

Our author gives us a definition of infection and contagion, of so great a length that they may be rather called descriptions than definitions. Among the diseases which are arranged under the head of Infection, we find putrid fevers, erysipelas, ophthalmia, &c. The distinctive property of infection is to have the power of affecting a great number of persons at the same time, without their having any communication with each other, or with those already labouring under the disease. In so far he agrees with his antagonists, that infectious diseases are not always or necessarily contagious; but to say that they never become so, he adds, is in direct opposition to the evidence of facts. In the instances which he adduces to prove this position, he places the true plague and the yellow fever upon the same level, and tells us, over and over again, the often-refuted stories of its propagation by importation at Cadiz, Xeres, Seville, &c. —nay, he affirms that the contagion is brought from the native country of the yellow fever, in the goods of the sick, in porous substances which they have touched, &c. &c. Thus we find that all the evidence which, in our mind, goes clearly to establish the non-contagious nature of the yellow fever, is either unknown to our author, or contemptuously rejected. His belief in contagion, indeed, is carried to an extent, of which we know no other example. "The list of contagious diseases, (he says,) both febrile and non-febrile, is sufficiently extensive, and we only pretended to speak of the first class here. Now, here are some of the incontestible circumstances under which they have been found to develop themselves."

The first example given is that of the Black-hole of Calcutta, in which, at the end of twenty-four hours, only twenty-three persons out of 146 were found alive.* These people, it is well known, died from the mere want of respirable air; and how this example can in any way favour M. Foderé's views, we are at a loss to understand.

He seems to think that the assembling together a number of sick persons is not always necessary to the production of a contagious disease, but that one diseased person alone, under favourable circumstances, will communicate a similar disease to

* In relating this example, M. Foderé makes more than one mistake. He states the number of persons confined to have been forty-five men and one woman, leaving out *only one hundred.* He extends their confinement to twenty-four hours; whereas they were imprisoned at eight in the evening, and the poor remainder were liberated at six o'clock on the following morning. Indeed, we should scarcely believe that M. Foderé was relating the same story, if the date and place did not compel us to do so.

any indefinite extent; and he quotes, apparently with approbation, from Professor CARMINATI, of Pavia, the history of a lying-in woman, otherwise healthy, who was shut up in a warm room, the air of which was not renewed, who was exposed to a heating regimen, and who contracted an acute and malignant fever, which became contagious. This is followed by an instance of a *miliary fever* succeeding to an ill-treated peripneumony, and which was communicated to several persons in the same family!

These extracts will, we trust, convince our readers how very unnecessary it is for us to pursue the examination of this part of M. Foderé's labours any further. We find throughout a great deal of reading, ill-digested, a large stock of credulity, and the most confirmed and bigotted prejudice to opinions, at least questionable on some points, and absolutely untenable on others.

The next chapter in succession (the sixth) is very short, and contains a *Classification of Epidemic Diseases according to their Causes*.—The divisions which he adopts are those diseases which are the product of bad food, of atmospheric intemperies, of marshy ground, of morbific effluvia which are borne in the air, those which arise from different principles of infection, and, lastly, those which are the productions of disease,—or, in other words, of contagion. "Certainly, (continues our author,) such an arrangement would not be despicable, and ought to be preferred, especially if it should unite the means of securing individuals, cities, and empires, with the knowledge of these diseases and their method of treatment: but when so many systems, invented to facilitate the study of natural history, have all their weak side; when all nosological arrangements have something forced and unnatural about them; when, quitting our books, where every thing is clear, and giving ourselves up to practice, we see the greater number of febrile diseases pass one into the other, either as a natural consequence or through error of treatment, we dare not present this classification but with great diffidence." (p. 241.)

We cannot but think the above arrangement extremely defective, and which an enumeration of the different orders will, we conceive, render obvious to our readers. Nor can we much applaud the precision or justice of many of the remarks which usher in this new classification; for example:

"*Typhus*, or *malignant fever*, (says M. Foderé,) very often arises from the elements which would seem only proper to give birth to *putrid* fevers; and, in an extended epidemic, one sees, among the sick, one or other of these fevers succeed to, and become complicated with, each other. As to the exanthemata, particularly such as are *symptomatic*, nothing is fixed or precise. *Erysipelas, petechiæ, purpura, miliary*

*eruptions, pemphigus*, &c. are often associated with diseases arising from all sorts of causes,—sometimes as signs of danger, sometimes as critical, and sometimes as insignificant appendages. Periodical fevers are not exempt. Swellings of the parotid glands, buboes, abscesses, carbuncular pustules, are not always necessarily signs of the true plague: they show themselves occasionally in typhoid fevers, in that of America, and in marsh fever. Finally, we have seen that the character of contagion cannot form one class, *because all diseases may acquire it;* and because, on the contrary, among the diseases *decidedly contagious*, there are some that, under certain circumstances, *are not communicated.*" (p. 243.)

We have printed some portion of this precious passage in italics, leaving our readers to form their own judgment of the clear conception and accurate knowledge of febrile diseases contained in these few lines.

We now proceed to enumerate the six orders of epidemic diseases, and their species. The first order, diseases arising from food or drink, contains six species,—simple gastric fever, worm fever, fever with convulsions or raphania, fever with gangrene from ergot, epidemic diarrhœa, dysentery, and dysenteric fever or scurvy. We shall only remark upon this order generally, that few of these diseases deserve to be classed among epidemics; and the causes assigned, in the instances of dysentery, worm-fever, and simple gastric fever, are mere assumptions, totally destitute of all proof.—The second order, or epidemics from miasmata, has three species only,—intermittent fevers, slow insidious fevers *(fièvres subintrantes et insidieuses)*, and remittent fevers, especially those of hot climates. Here we find that the yellow fever does not find its place, although as clearly the product of marsh miasmata as either of the foregoing species; but this will be afterwards found, not only in one, but in two successive orders, first as a disease the product of infection, and, secondly, among those called contagious.—The third order contains eight species; it is entitled, Diseases arising solely from variations in the atmosphere: these are inflammatory fevers, inflammations, bilious and ardent bilious fevers, cholera morbus, colics, catarrhal fevers, mucous, pituitous, and mesenteric fevers, colds and pulmonary catarrh, hooping-cough, and croup. Here again we have abundance of instances of gratuitous assumptions: can M. Foderé positively affirm that either hooping-cough, or cholera morbus, or affections of the mesentery, are dependent solely upon atmospheric changes? To us it appears that, if the names of these diseases had been drawn by lottery, they could scarcely be arranged in a less scientific or more apparently fortuitous manner.—The fourth order of diseases, arising from heterogeneous substances transported in the air, is equally fanciful: here are six species of ophthalmia, epidemic gangrenous angina, (what is that?) false pleurisies, or peripneumony, epidemic miliary fevers and sweats, puerperal fevers, and erysipelatous fevers.—The fifth order comprises diseases of infection, and contains four species: the putrid fever, yellow fever of the equinoctial regions of America, the epidemic petechial fever, and epidemic malignant pustule of hospitals. Here we find it decided that petechial and putrid fevers are distinct from each other, as also from the true typhus; that the yellow fever of America is, in that country, only the produce of infection. And the sixth order, containing the contagious diseases, in number seven,—namely, the European typhus, the plague, or Eastern typhus, the imported yellow fever, the small-pox, measles, scarlet fever, and *epidemic syphilis*, completes this climax of absurdity: for, by this arrangement, M. Foderé decides that the yellow fever is always an imported malady in Europe, since he has struck it out of the order of diseases from infection. He has done so also by small-pox, measles, and scarlet fever; though there can be no reason whatever for doubting that these complaints, although undoubtedly contagious, or to all intents and purposes the product of a particular infectious condition of the atmosphere; and is not that proved by these diseases being epidemic only in some particular seasons or at some particular places, whilst at other times sporadic cases only are to be met with; the contagious quality of such cases remaining in equal force.

The first section of the volume concludes with chapter viith, being a general view of the *means of preventing diseases.*—We may dismiss this chapter with one observation only: it contains nothing new, but, generally speaking, the rules and cautions recommended are judicious. Our author, placing the yellow fever and the plague upon the same footing, advocates the strict enforcement of the quarantine laws in both instances. In the case of the plague, we most cordially agree with him, and refer our readers to some highly judicious, but rather severe strictures, which Dr. Paris* has published on the work of Dr. Maclean; a work to which may be applied the remark of Holofernes,† "that he draweth out the web of his verbosity finer than the staple of his argument." With regard to the rigid application of quarantine to the case of yellow fever, many reasonable objections may be urged: it may be highly proper, in the present state of our knowledge, to exact a strict scrutiny into all vessels arriving from ports where that disease is known to rage; it may be quite necessary to segregate those who arrive with suspicious symptoms from the sound and healthy; but surely, when the local origin of this fever is made out almost to

* *Medical Jurisprudence,* vol. i. † *Love's Labour Lost.*

a demonstration, to shut up the wretched inhabitants in the very focus of infection,—to draw a line round a multitude of our fellow creatures, and thereby to devote them to that destruction which a removal from the origin of the pestilence would altogether prevent, is a most barbarous policy, and one which all recent examples show to be not only cruel, but totally unnecessary, and even futile.

---

### STATISTICAL MEDICINE.

